# From belief to resilience: mapping the longitudinal path of Traditional Paranormal Belief through optimism and Mental Toughness

**DOI:** 10.3389/fpsyg.2026.1856551

**Published:** 2026-09-28

**Authors:** Neil Dagnall, Andrew Denovan, Robert Dempsey, Kenneth Graham Drinkwater, Álex Escolà-Gascón, Stephen Walsh

**Affiliations:** 1 School of Psychology, Manchester Metropolitan University, Manchester, United Kingdom; 2 School of Psychology, Liverpool John Moores University, Liverpool, United Kingdom; 3 Blanquerna Foundation, Ramon Llull University, Barcelona, Spain

**Keywords:** Mental Toughness, New Age Philosophy, optimism, psychological well-being, stress, Traditional Paranormal Belief

## Abstract

**Introduction:**

Although researchers have traditionally focused on the irrational nature of paranormal belief (PB), contemporary perspectives suggest it can serve adaptive psychological functions, including meaning-making and perceived control. However, the mechanisms through which PB relates to positive psychological functioning remain unclear. Noting this, the present study examined whether Traditional Paranormal Belief (TPB) and New Age Philosophy (NAP) influenced stress and well-being indirectly via optimism and Mental Toughness.

**Method:**

The study used a multiple time point design. 1,032 United Kingdom adults were assessed across three waves spaced four to 6 weeks apart. Participants completed measures of PB (TPB, NAP), optimism, Mental Toughness, perceived stress, and hedonic and Eudaimonic Well-Being. Using longitudinal path analysis with bootstrapped indirect effects, analysis tested two three-wave autoregressive mediation models.

**Results:**

TPB (not NAP) predicted outcomes indirectly through a sequential pathway involving optimism and Mental Toughness. Specifically, TPB was associated with lower stress and higher hedonic well-being via these mediators. Direct effects of PB on outcomes were small and inconsistent. Across models, optimism positively predicted Mental Toughness, which predicted lower stress and higher levels of hedonic and Eudaimonic Well-Being.

**Discussion:**

Findings indicate that PB operates as a distal cognitive framework that influences psychological functioning through indirect pathways. In particular, TPB appears to foster optimistic appraisals and strengthen Mental Toughness, which support resilience and well-being over time. These results demonstrate that the psychological value of PB lies in its role in mobilising psychological resources rather than exerting direct effects on outcomes.

## Introduction

Although characterised by critics as non-scientific or irrational, due to the absence of objective empirical support (Dagnall et al., 2026), recent research has reconceptualised paranormal belief (PB) as a facet of human cognition that can serve positive psychological functions (i.e., afford meaning, coherence, and perceived control) (Dagnall et al., 2025a,b). These adaptive features explain why PB prevails within modern Western societies and is endorsed by a substantial proportion of the general population (Irwin, 2009). Cognizant of this, the present paper operationalised PB as validation of propositions (e.g., powers, forces, and entities) generated by non-scientific communities that people capable of rational thought and reality testing ratify, despite lack of scientific authentication (Dagnall et al., 2016; Irwin, 2009).

This definition aligns with the conceptual underpinnings of the Revised Paranormal Belief Scale (RPBS, Tobacyk, 2004), which represents PB as a multidimensional construct encompassing seven domains (i.e., psi, anomalous mental processes; witchcraft, magical agency; superstition, luck and fate; spiritualism, separation of consciousness from the physical body and afterlife communication; extraordinary life form, unidentified biological entities; and precognition, foreknowledge of events). The circumscribing feature of these phenomena being violation of current scientific principles (Broad, 1949).

Subsequent research has questioned the consistency and invariance of RPBS dimensions across samples (see Drinkwater et al., 2017). While this debate sits outside of the remit of this paper, a pertinent measurement advance was the identification of a psychometrically superior, two-factor model comprising Traditional Paranormal Belief, TPB; and New Age Philosophy, NAP. This model categorizes beliefs based on psychological function rather than content. TPB indexes culturally transmitted and externally validated beliefs (e.g., religious or supernatural doctrines), whereas NAP captures experiential, internally derived beliefs (e.g., spirituality and psychic phenomena).

TPB provides a sense of order by attributing life events to social/cultural supernatural forces (e.g., god, fate, witchcraft) (Goode, 2000). This facilitates coping by suggesting that even when personal agency is absent, a higher power or force remains in control. NAP affords a sense of agency through internalized abilities (e.g., psi, healing, meditation) (Irwin, 1992). This factor emphasizes the individual’s capacity to influence reality through spiritual or psychic means (Drinkwater et al., 2024).

The divergent functions of TPB and NAP are reflected in their distinct relationships with psychopathology. Houran et al. (2001) reported that NAP showed small consistent associations with pathological and nonpathological dissociation (i.e., a sense of detachment) and the perceptual-cognitive factor of schizotypy. These schizotypal characteristics, resemble attenuated positive-productive, psychotic symptoms (e.g., magical thinking and unusual perceptions), which though distinct from reality, remain within the non-clinical range of the general population.

Despite the emergence of the two-factor model, researchers have continued to utilize total RPBS scores. While a global score offers a sound, measure of belief prevalence, which is useful for quantifying individual differences, it fails to differentiate between the divergent motivations and functions associated with belief types. This is why the application of the two-factor model to well-being and related outcomes is academically beneficial. The principal advantage being enhanced interpretability of findings. For instance, the two-factor model may help to clarify mixed results reported by prior studies assessing relationships between PB and psychological functioning. Particularly, why some investigations report that PBs are affiliated with maladaptive outcomes (i.e., increased anxiety and stress) (e.g., Lasikiewicz, 2016), whereas other indicate potential benefits (i.e., enhanced coping or subjective well-being) (e.g., Dagnall et al., 2025b).

The comprehension of inconsistencies is further compounded by methodological limitations, which obscure the true nature of associations between PB and well-being. Particularly, reliance on cross-sectional designs prevents determination of variable temporal ordering, leaving it unclear whether belief systems shape psychological states or reflect affective states. Additionally, researcher focus on direct associations ignores the indirect mechanisms through which beliefs exert influences (Denovan et al., 2020; Swami et al., 2016). This is an important consideration because beliefs, as a function of being embedded within cognitive and motivational processes, may operate as distal predictors. In this capacity, they exert an effect through intermediary pathways, such as cognitive appraisals and/or coping resources.

The failure to account for latent, longitudinal, and indirect effects explains why observed relationships between PB and well-being remain theoretically underspecified. To address these conceptual and methodological gaps, the present study progressed beyond direct associations to examine the functional pathways through which PB systems operate. By mapping indirect longitudinal pathways, this study demonstrates how PB systems facilitate resource mobilization. This challenges the deficit model by reframing PB within a functional clinical resilience framework.

### Present study

While TPB and NAP differentially relate to psychological functioning, both are conceptualized as cognitive frameworks that individuals employ to interpret uncertainty and derive meaning from experience. Consequently, their influence involves multi-stage processes rather than direct effects. A primary candidate for this intermediary role, bridging belief and well-being, is optimism. By providing a sense of coherence or perceived control in unpredictable contexts, TPB and NAP may foster optimistic expectations, even when beliefs lack empirical grounding (Dagnall et al., 2025b; Irwin, 2009). In turn, heightened optimism is a robust predictor of adaptive coping and enhanced psychological well-being (Carver et al., 2010; Scheier and Carver, 1985).

Beyond immediate affective benefits, optimism is associated with the cultivation of stable psychological resources. Specifically, optimism is a significant predictor of Mental Toughness, a construct reflecting resilience, perseverance, and the ability to maintain performance under stress (Clough et al., 2002). Individuals reporting higher levels of optimism tend to engage in active coping strategies and persist in the face of challenges, which is theoretically linked to the development of Mental Toughness (Nicholls et al., 2008).

Mental Toughness, in turn, is strongly associated with lower perceived stress and higher levels of hedonic and Eudaimonic Well-Being (Lin et al., 2017). This suggests a theoretically coherent sequential pathway in which beliefs shape cognitive appraisals (optimism), which build psychological resources (Mental Toughness), improving well-being outcomes.

Importantly, previous research suggests that PB, including TPB and NAP, show weak and inconsistent direct associations with well-being outcomes (Dagnall et al., 2022a,b, 2024). As such, the influence of PB is likely to operate indirectly through intermediary psychological processes rather than as a direct determinant of outcomes. Examining indirect pathways thus provides a sensitive and theoretically meaningful test of how PB types relate to psychological functioning over time (Preacher, 2015).

Based on this framework, it was hypothesised that (H1) TPB and NAP at Time 1 would positively predict optimism at Time 2; (H2) optimism at Time 2 would positively predict Mental Toughness at Time 2; and (H3) Mental Toughness at Time 2 would predict lower stress and higher hedonic and Eudaimonic Well-Being at Time 3. Furthermore, (H4) TPB and NAP would exert indirect effects on psychological outcomes via optimism and Mental Toughness over time. Given the mixed evidence regarding direct effects, no strong predictions were made regarding direct associations between PB and later well-being outcomes.

Although cross-lagged panel models (CLPMs) are commonly used to examine reciprocal longitudinal associations, recent methodological work has highlighted important limitations of fully saturated cross-lagged models, including overparameterization, reduced statistical power, and difficulties in interpretation when large numbers of cross-lagged paths are estimated simultaneously (Kline, 2016; Little, 2013; Selig and Little, 2012). In particular, including all possible cross-lagged effects without theoretical justification can obscure meaningful relationships and increase the risk of identifying spurious or trivial effects (Cole and Maxwell, 2003; MacCallum, 1995).

In line with recommendations for theory-driven model specification, the present study adopted a parsimonious longitudinal mediation framework, specifying only cross-lagged paths that were theoretically grounded (Little, 2013; Newsom, 2015). Specifically, the model focused on a hypothesised directional process, whereby PB influences optimism, which in turn predicts Mental Toughness, and subsequently psychological outcomes. This approach precisely tests proposed mechanisms while maintaining adequate statistical power and interpretability (Kline, 2016; Preacher, 2015).

Furthermore, by incorporating autoregressive and long-lag stability paths, the model controls for prior levels of all constructs, thereby providing a stringent test of longitudinal change and reducing bias associated with omitted stability effects (Cole and Maxwell, 2003). Together, this approach reflects current best practice in longitudinal path modelling, prioritising parsimony, theoretical coherence, and interpretability, over fully saturated cross-lagged designs (Little, 2013; Orth et al., 2021). See Figure 1 for a schematic representation of the hypothesised model for TPB (NAP was assessed using the same approach/model structure).

**Figure 1:**
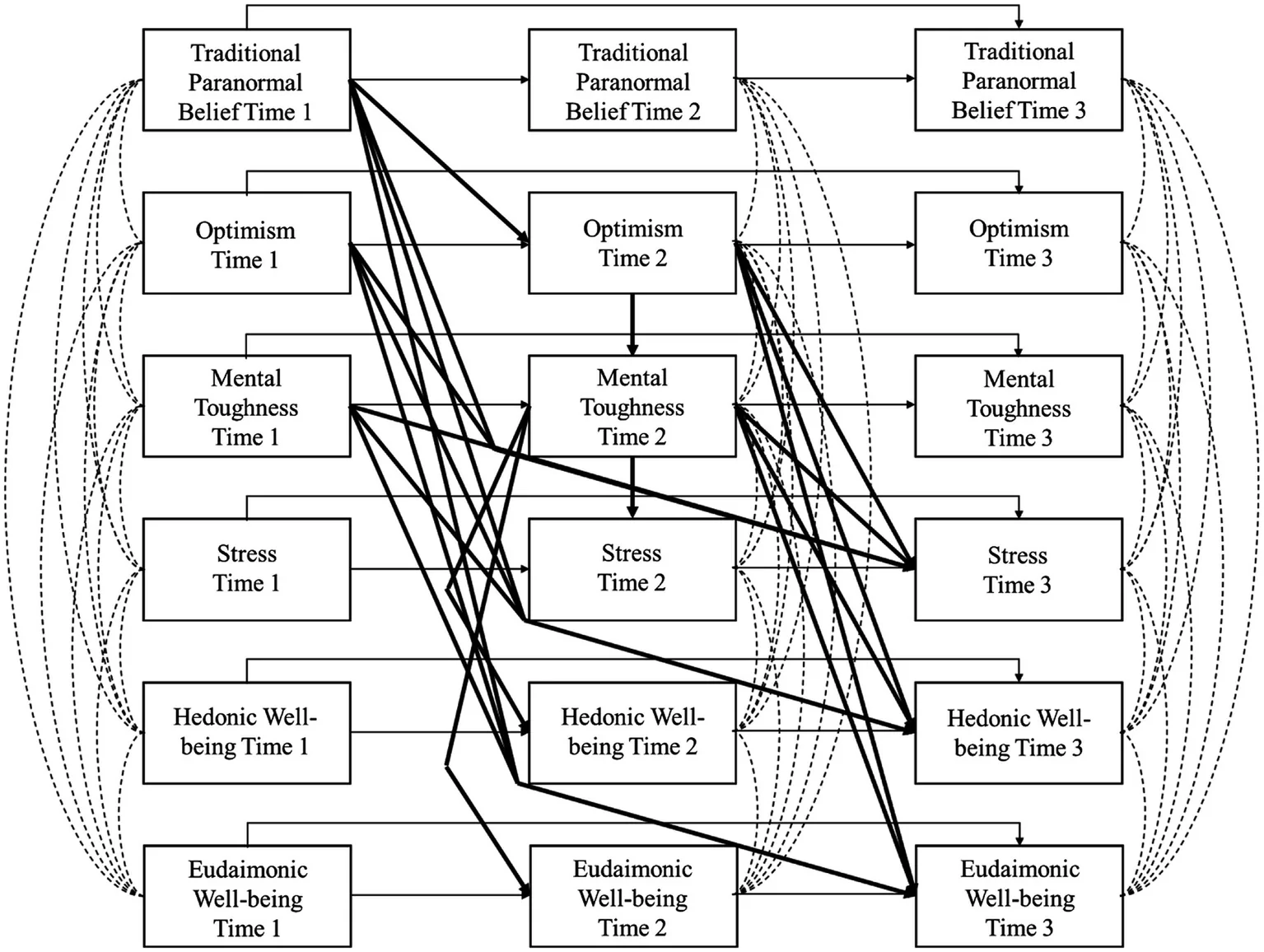
Schematic representation of three-wave autoregressive mediation model with Traditional Paranormal Beliefs (Model 1). Dotted lines represent within-wave correlations, bold lines represent cross-lagged paths, non-bold solid lines represent autoregressive paths.

## Method

### Participants

The sample comprised 1,032 United Kingdom adults recruited via Bilendi, an acknowledged provider of high-quality research participants (De Coninck et al., 2021). Data were collected across three waves, 4–6 week apart. Participants provided complete data at all three time points. At Time 1, participants ranged in age from 18 to 79 years (M = 52.92, SD = 13.83), with comparable age distributions observed at Time 2 (M = 52.97, SD = 13.84) and Time 3 (M = 53.08, SD = 13.81), indicating stability across waves.

The gender distribution consisted of 517 men (50.1%; M = 54.37, SD = 13.15), 511 women (49.5%; M = 51.53, SD = 14.34), and 4 participants identifying as non-binary (0.4%). The breakdown of educational attainment (Time 1) was 214 participants (20.7%) pre-degree qualifications, 300 (29.1%) undergraduate degrees, 191 (18.5%) postgraduate degrees, 205 (19.9%) vocational qualifications, and 122 (11.8%) professional qualifications. Age varied across qualification levels, with higher qualification categories associated with older mean age (e.g., pre-degree: M = 58.51, SD = 12.12; undergraduate: M = 48.22, SD = 13.69).

### Measures

#### Paranormal belief

The Revised Paranormal Belief Scale (RPBS; Tobacyk, 2004) measured endorsement of supernatural powers and entities. The scale contains 26 items, presented as statements (e.g., ‘There is a devil’) and participants respond using a 7-point Likert-type scale (1 = Strongly Disagree to 7 = Strongly Agree). Following Rasch scaling instructions the researchers recoded scores 0 to 6 and produced transformed scores for Traditional Paranormal Belief (TPB, 5 items) (encompassing Witchcraft, Superstition, and Spiritualism) and New Age Philosophy (NAP, 11 items) (embracing psi, Extraordinary Life Forms, and Precognition). Higher subscales scores indicate greater belief.

#### Optimism

The Optimism–Pessimism Short Scale–2 (SOP2) (Kemper et al., 2013; Nießen et al., 2022) evaluates dispositional confidence and future expectations. This study used only the optimism item (i.e., ‘How optimistic are you in general’). Participants recorded their answers on a 7-point Likert-type scale (1 = not at all to 7 = very). Higher scores indicate greater optimism about the future.

#### Mental toughness

The Mental Toughness Questionnaire-10 (MTQ10; Dagnall et al., 2019; Papageorgiou et al., 2018) evaluates positive mindset and the ability to perform optimally under pressure. The scale comprises 10 items, which appear as statements (e.g., ‘I generally feel in control’). Participants respond using a 5-point Likert-type scale (1 = Strongly Disagree to 5 = Strongly Agree). Higher totals designate greater Mental Toughness.

#### Stress

The Single-Item Stress Question (SISQ; Elo et al., 2003) assessed stress symptoms. The item provides a brief definition of stress and asks participants to indicate the extent to which they experience symptoms. Responses are recorded on a 5-point Likert -type scale ranging from 1 (not at all) to 5 (very much). Higher scores indicate greater perceived stress.

#### Psychological well-being

The Eudaimonia and Hedonia Scale (Su et al., 2014) assessed well-being. The eudaimonic subscale (6 items) assessed positive psychological functioning and personal growth, while hedonic wellbeing subscale (5 items) assessed life satisfaction and general happiness. Within the scale items appear as statements (e.g., ‘I have a sense of purpose in my life’). Participants respond via a 7-point Likert-type scale (1 strongly disagree to 7 completely agree). Greater eudaimonic and hedonic well-being is indicated by higher subscale totals.

All measures possessed verified psychometric properties (i.e., validity and reliability): RPBS (Drinkwater et al., 2017), SOP2 (Kemper et al., 2013; Nießen et al., 2022), MTQ10 (Dagnall et al., 2019), SISQ (Elo et al., 2003), and Eudaimonia and Hedonia Scale (Su et al., 2014). Internal consistency for multi-item measures was acceptable across time points (Cronbach’s *αs* ≥ 0.70). As SOP2 and SISQ were single-item measures, internal consistency coefficients were not applicable. However, prior research supports the reliability and validity of these indicators.

#### Procedure

Potential participants received a web link inviting them to take part in the study. Activating the link enabled access to the participation information sheet (PIS) hosted in Qualtrics. This outlined study aims and ethics. Consenting participants then progressed to the demographic information (always presented first) then the self-report measures. To control for potential order effects, the sequence of sections was randomized by Qualtrics.

Given the cross-sectional nature of the data collection, the researchers implemented methodological strategies to reduce common-method variance. In line with established protocols, survey instructions emphasized section independence (Podsakoff et al., 2003). This created psychological separation between constructs, thereby reducing the likelihood of uniform response patterns (Krishnaveni and Deepa, 2013). To alleviate evaluation apprehension and social desirability bias, the study brief explicitly stated that responses should reflect personal perspectives. This strategy encourages transparent and authentic participant input (Denovan et al., 2024, 2025). All participants who completed the initial survey, were aware the researchers would invite them to complete subsequent time points (T2 and T3). Nonetheless, the procedure on each occasion remained the same (i.e., the researchers provided participants with the PIS and they recorded informed consent).

#### Analysis

Data were analysed using longitudinal path modelling in Mplus version 8 (Muthén and Muthén, 2018). Two three-wave autoregressive mediation models were specified to examine whether Traditional Paranormal Beliefs (TPB, Model 1) and New Age Philosophy (NAP, Model 2) predicted changes in stress and positive psychological outcomes (hedonic and Eudaimonic Well-Being) over time via optimism and Mental Toughness.

Models included autoregressive (stability) paths for variables across adjacent time points (Time 1 → Time 2 → Time 3), as well as long-lag stability paths from Time 1 to Time 3. Including these paths controls for prior levels of each construct, allowing for the examination of residual change over time (Cole and Maxwell, 2003). The hypothesised mediation models specified that TPB and NAP at Time 1 predicted optimism at Time 2, optimism at Time 2 predicted Mental Toughness at Time 2, and Mental Toughness at Time 2 predicted psychological outcomes (i.e., stress, hedonic well-being, and Eudaimonic Well-Being). In addition, direct effects from Time 1 predictors (TPB, NAP, optimism, and Mental Toughness) to Time 3 outcomes were included to test for partial mediation.

Models incorporated cross-lagged paths based on theoretical expectations rather than a fully saturated model. This approach was consistent with recommendations to prioritise theory-driven model specification and parsimony in longitudinal structural models (Kline, 2016). Within each time point, variables were allowed to covary freely to account for concurrent associations among constructs. This approach reduces bias by controlling shared variance at each wave (Little, 2013).

Models incorporated maximum likelihood estimation to enable use of bootstrapping for indirect effects. Missing data were handled using full information maximum likelihood (FIML), which uses all available data under the assumption of missing at random (MAR) and is recommended for longitudinal analyses (Enders, 2010). Model fit was evaluated using multiple indices, including Comparative Fit Index (CFI), Tucker–Lewis Index (TLI), Root Mean Square Error of Approximation (RMSEA), and Standardized Root Mean Square Residual (SRMR). Following conventional guidelines, values of CFI and TLI ≥ 0.90 designate acceptable fit, while RMSEA ≤ 0.08 and SRMR ≤ 0.08 indicate adequate model fit (Hu and Bentler, 1999).

Indirect effects were tested using bias-corrected bootstrap confidence intervals (1,000 resamples). Bootstrapping is recommended for mediation analysis because it does not assume normality of the indirect effect distribution (Preacher and Hayes, 2008). Significant indirect effects were identified when the 95% confidence interval did not include zero. This modelling approach allowed for a stringent test of longitudinal mediation while controlling for prior levels of all variables, thereby providing a conservative estimate of the prospective effects of paranormal beliefs on psychological outcomes.

## Results

### Descriptive statistics and correlations

Descriptive statistics for study variables across three time points appear in Table 1. Mean levels were stable across time. For example, Optimism remained consistent from Time 1 (M = 4.37, SD = 1.51) to Time 3 (M = 4.39, SD = 1.54), and Mental Toughness showed minimal variation (Time 1: M = 33.71, SD = 7.28; Time 3: M = 34.01, SD = 7.22). Similarly, Perceived Stress remained stable (Time 1: M = 2.81, SD = 1.23; Time 3: M = 2.81, SD = 1.23).

**Table 1:** Descriptive statistics for study variables across three waves.

Variable	Time 1 M (SD)	Time 2 M (SD)	Time 3 M (SD)
Traditional Paranormal Belief	22.63 (6.23)	22.77 (6.20)	22.54 (6.42)
New Age Philosophy	21.63 (6.77)	21.60 (6.72)	21.31 (6.89)
Optimism	4.37 (1.51)	4.32 (1.54)	4.39 (1.54)
Mental Toughness	33.71 (7.28)	33.74 (7.31)	34.01 (7.22)
Stress	2.81 (1.23)	2.88 (1.23)	2.81 (1.23)
Hedonic Well-Being	43.55 (8.03)	42.28 (7.97)	39.71 (7.69)
Eudaimonic Well-Being	29.21 (6.52)	29.37 (6.58)	29.19 (6.48)

SD, standard deviation.

Zero-order correlations for Time 1 exist in Table 2 (see Supplementary Tables 1, 2 for Time 2 and 3 correlations). Strong temporal stability was observed for constructs. For example, TPB and NAP showed high autoregressive correlations across waves (*rs* ≈ 0.82–0.88, *ps* < 0.001), and Optimism (*rs* ≈ 0.75–0.77), Mental Toughness (*rs* ≈ 0.87–0.89), and Stress (*rs* ≈ 0.69–0.70) were also highly stable. Importantly, the pattern of associations at Time 1 was highly consistent across Time 2 and 3.

**Table 2:** Correlations among time 1 study variables.

Variable	1	2	3	4	5	6	7
1. Traditional Paranormal Belief	—						
2. New Age Philosophy	0.81^**^	—					
3. Optimism	0.15^**^	0.15^**^	—				
4. Mental Toughness	−0.08^*^	−0.10^**^	0.54^**^	—			
5. Stress	0.18^**^	0.18^**^	−0.31^**^	−0.60^**^	—		
6. Hedonic Well-Being	−0.06	−0.09^**^	0.48^**^	0.70^**^	−0.45^**^	—	
7. Eudaimonic Well-Being	0.07^*^	0.05	0.61^**^	0.67^**^	−0.40^**^	0.78^**^	—

^*^p < 0.05, ^**^p < 0.001.

Cross-sectionally, Optimism was positively associated with well-being indicators, including Eudaimonic Well-Being (*r* = 0.61, *p* < 0.001), Hedonic Well-being (*r* = 0.48, *p* < 0.001), and Mental Toughness (*r* = 0.54, *p* < 0.001). Stress was negatively associated with these constructs (e.g., Mental Toughness: *r* = −0.60, *p* < 0.001). TPB and NAP showed weak associations with positive psychology constructs.

### Longitudinal path models

To examine whether paranormal belief types (TPB and NAP) predicted changes in optimism, Mental Toughness, well-being, and stress over time analysis tested two longitudinal path models. The model for TPB (Model 1) demonstrated acceptable data fit, *χ*^2^(77) = 749.18, *p* < 0.001, CFI = 0.96, TLI = 0.93, RMSEA = 0.09, 90% CI [0.08, 0.09], SRMR = 0.06. Regarding structural paths, constructs demonstrated strong temporal stability (*βs* = 0.53–0.82, *ps* < 0.001). See Table 3.

**Table 3:** Standardized structural path estimates for Model 1 (TPB).

Pathway	*β*	*p*
Stability effects		
T1 Traditional Paranormal Belief → T2 Traditional Paranormal Belief	0.82	<0.001
T2 Traditional Paranormal Belief → T3 Traditional Paranormal Belief	0.53	<0.001
T1 Traditional Paranormal Belief → T3 Traditional Paranormal Belief	0.40	<0.001
T1 Optimism → T2 Optimism	0.74	<0.001
T2 Optimism → T3 Optimism	0.46	<0.001
T1 Optimism → T3 Optimism	0.41	<0.001
T1 Mental Toughness → T2 Mental Toughness	0.79	<0.001
T2 Mental Toughness → T3 Mental Toughness	0.52	<0.001
T1 Mental Toughness → T3 Mental Toughness	0.42	<0.001
T1 Stress → T2 Stress	0.53	<0.001
T2 Stress → T3 Stress	0.33	<0.001
T1 Stress → T3 Stress	0.33	<0.001
T1 Hedonic Well-Being → T2 Hedonic Well-Being	0.56	<0.001
T2 Hedonic Well-Being → T3 Hedonic Well-Being	0.41	<0.001
T1 Hedonic Well-Being → T3 Hedonic Well-Being	0.35	<0.001
T1 Eudaimonic Well-Being → T2 Eudaimonic Well-Being	0.78	<0.001
T2 Eudaimonic Well-Being → T3 Eudaimonic Well-Being	0.36	<0.001
T1 Eudaimonic Well-Being → T3 Eudaimonic Well-Being	0.35	<0.001
Cross-lagged effects		
T1 Traditional Paranormal Belief → T2 Optimism	0.05	0.024
T2 Optimism → T3 Stress	−0.01	0.750
T2 Optimism → T3 Hedonic Well-Being	0.01	0.767
T2 Optimism → T3 Eudaimonic Well-Being	0.08	0.022
T2 Mental Toughness → T3 Stress	−0.15	0.006
T2 Mental Toughness → T3 Hedonic Well-Being	0.08	0.035
T2 Mental Toughness → T3 Eudaimonic Well-Being	0.05	0.212
Direct Effects (T1 → T3)		
T1 Traditional Paranormal Belief → T3 Stress	0.04	0.065
T1 Traditional Paranormal Belief → T3 Hedonic Well-Being	0.01	0.459
T1 Traditional Paranormal Belief → T3 Eudaimonic Well-Being	0.02	0.194
T1 Mental Toughness → T3 Stress	−0.08	0.125
T1 Mental Toughness → T3 Hedonic Well-Being	0.11	0.006
T1 Mental Toughness → T3 Eudaimonic Well-Being	0.10	0.012
T1 Optimism → T3 Stress	−0.04	0.277
T1 Optimism → T3 Hedonic Well-Being	0.01	0.826
T1 Optimism → T3 Eudaimonic Well-Being	0.04	0.280
Indirect Effects (T1 → T3 via T2 Variables)		
T1 Traditional Paranormal Belief → T2 Optimism → T3 Stress	−0.009	0.060
T1 Traditional Paranormal Belief → T2 Optimism → T2 Mental Toughness → T3 Stress	−0.01	0.044
T1 Traditional Paranormal Belief → T2 Optimism → T3 Hedonic Well-Being	0.008	0.082
T1 Traditional Paranormal Belief → T2 Optimism → T2 Mental Toughness → T3 Hedonic Well-Being	0.01	0.046
T1 Traditional Paranormal Belief → T2 Optimism → T3 Eudaimonic Well-Being	0.004	0.133
T1 Traditional Paranormal Belief → T2 Optimism → T2 Mental Toughness → T3 Eudaimonic Well-Being	0.001	0.926

Values are standardized coefficients (*β*). Traditional Paranormal Belief was assessed across three time points. All paths are presented in forward temporal order (T1 → T2 → T3). Models were estimated using maximum likelihood with bootstrapped standard errors.

TPB at T1 showed no consistent direct effects on Time 3 outcomes. The direct path to Stress was significant (*β* = 0.04, *p* = 0.046), but non-significant for Hedonic Well-being (*β* = 0.01, *p* = 0.845), and Eudaimonic Well-Being (*β* = 0.05, *p* = 0.128). Optimism and Mental Toughness represented key predictors. Optimism positively predicted Mental Toughness at Time 2 (*β* = 0.14, *p* < 0.001), and Mental Toughness predicted lower stress (*β* = −0.18, *p* < 0.001), higher Hedonic Well-being (*β* = 0.17, *p* < 0.001), and higher Eudaimonic Well-Being (*β* = 0.14, *p* < 0.001).

No significant indirect effects existed with regards to Optimism. However, statistically significant indirect effects on Stress and Hedonic well-being were observed through a sequential pathway involving Optimism and Mental Toughness (*β* = −0.01, *p* = 0.043 and *β* = 0.01, *p* = 0.046). This provides pathway-specific evidence for mediation. No significant indirect effects existed in relation to Eudaimonic Well-Being.

The NAP model (Model 2) demonstrated acceptable data fit, *χ*^2^(77) = 737.71, *p* < 0.001, CFI = 0.96, TLI = 0.93, RMSEA = 0.09, 90% CI [0.08, 0.09], SRMR = 0.06. All constructs again showed strong temporal stability concerning structural paths (*βs* = 0.53–0.82, *ps* < 0.001). See Supplementary Table 3 for results. Critically, NAP at T1 did not significantly predict Optimism at Time 2 (*β* = 0.01, *p* = 0.557), nor any Time 3 outcomes directly (*βs* = 0.003–0.02, *ps* > 0.43). Since the initial path in the mediation chain was non-significant, the hypothesised mediation pathway was not supported. As in Model 1, Optimism and Mental Toughness were the primary drivers of outcomes. Optimism predicted Mental Toughness (*β* = 0.14, *p* < 0.001), and Mental Toughness predicted lower Stress and higher well-being across time. Bootstrapped analyses indicated no significant indirect effects from NAP at T1 to any outcomes (all *ps* > 0.61), confirming the absence of mediation.

These findings reveal that PBs only drive minimal changes in positive psychological functioning, whereas established psychological resources (Optimism and Mental Toughness) play a leading role.

## Discussion

The present study examined whether Traditional Paranormal Beliefs (TPB) and New Age Philosophy (NAP) predicted stress and positive psychological well-being over time via optimism and Mental Toughness. Although direct paranormal beliefs (PB) effects on outcomes were small and inconsistent (Dagnall et al., 2024, 2025a,b), findings provided evidence for longitudinal indirect pathways. This aligned with recent research, which has demonstrated that PB influences meaning in life through the mediation of self-esteem and creativity (Dagnall et al., 2025a).

TPB predicted lower Stress and higher Hedonic Well-being through a sequential process involving Optimism and Mental Toughness. This suggests that because TPB derives from culturally transmitted and externally validated doctrines, it provides a sense of order that facilitates coping and positivity even in the absence of personal agency (Dagnall et al., 2025b; Irwin, 2009). This reflects TPB’s capacity to attribute life events to external forces, providing coherence in unpredictable contexts.

In contrast, the non-significant NAP model implies that internally generated beliefs lack the psychological stability required to mobilize the same resources as TPB. This is because NAP encompasses experiential phenomena associated with dissociation and the perceptual-cognitive factor of schizotypy (i.e., magical thinking and unusual perceptions) (Houran et al., 2001). Although NAP ideations are within the non-clinical range, they fail to stabilize the psychological resources necessary for the longitudinal mediation observed with TPB.

Across models, Optimism and Mental Toughness emerged as mechanisms of change, with Optimism positively predicting Mental Toughness and Mental Toughness, in turn, predicting lower stress and higher Well-being. This confirms the theoretical role of optimism as a robust predictor of adaptive coping (Carver et al., 2010; Scheier and Carver, 1985) and its link to Mental Toughness, which reflects the ability to maintain performance under stress (Clough et al., 2002; Nicholls et al., 2008). This pattern is also consistent with emerging work on non-cognitive adaptive resourcefulness (NCAR), which conceptualises constructs such as Mental Toughness, resilience, and self-efficacy as a broader system of dispositional resources underpinning adaptive functioning (Denovan et al., 2023a,b, 2026).

These findings indicate that PBs influence psychological well-being subtly and indirectly, as a distal predictor operating by strengthening psychological resources. Moreover, though TPB predicted lower Stress and higher Hedonic Well-being, the absence of a significant pathway to Eudaimonic Well-Being suggests the sense of control provided by external supernatural forces facilitates immediate stress reduction and life satisfaction rather than personal growth and purposeful agency.

### Limitations

The authors acknowledge that the study possessed limitations. Although the longitudinal framework with autoregressive controls provided a more stringent test of temporal precedence than cross-sectional designs, it did not definitively establish causality. Accordingly, future research should employ experimental or intervention-based designs to further explore how PB systems directly influence the development of psychological resources over time.

A further consideration was the 4–6 week time lag between waves. While the authors selected this interval to capture short-term shifts in psychological states, it is possible that the influence of deeply held belief systems on dispositional resources operates differently over longer periods. To test this, future research should vary intervals to determine if indirect pathways stabilize or fluctuate across differing time windows.

Another limitation was the use of single-item measures (e.g., SOP2 and SISQ). While these instruments are psychometrically sound and frequently feature in large-scale longitudinal research, they lack the construct breadth and dimensionality of longer scales. Hence, proceeding research would benefit from employing multidimensional scales to capture the nuances of stress, dispositional optimism, and well-being. For instance, inclusion of the Life Orientation Test-Revised (Scheier et al., 1994) would provide a distinction between the promotion of optimism and the mitigation of pessimism. Similarly, incorporation of the Perceived Stress Scale (PSS; Cohen et al., 1983; Cohen and Williamson, 1988), by delineating between perceived helplessness and perceived self-efficacy, would determine whether PB reduces the sense of being overwhelmed (perceived helplessness) or actively bolsters cognitive mechanisms involved in resource mobilization (perceived self-efficacy).

Regarding findings generalizability, the sample was restricted to United Kingdom adults. Given that TPB is heavily influenced by cultural and religious transmission, the observed pathways may differ in cultures with different supernatural doctrines or higher levels of secularization. Hence, cross-cultural validation is necessary to determine if TPB functions consistently across populations.

Finally, while the two-factor model distinguished between TPB and NAP, PB is a broad construct. Thus, future research should investigate whether specific sub-facets of belief contribute differently to the mobilization of resources. Understanding distinctions would clarify the conditions under which supernatural frameworks serve as adaptive cognitive mechanisms.

## Data Availability

The raw data supporting the conclusions of this article will be made available by the authors, without undue reservation.
