# Supplementary material for: From belief to resilience: mapping the longitudinal path of Traditional Paranormal Belief through optimism and Mental Toughness
Source: Front Psychol. 2026 Sep 28;17:1856551. doi: 10.3389/fpsyg.2026.1856551 (PMC13654991; doi:10.3389/fpsyg.2026.1856551)
Supplement: Supplementary file 1 [file Supplementary_file_1.docx]

Supplementary Table 1. Correlations Among Time 2 Study Variables

| Variable | 1 | 2 | 3 | 4 | 5 | 6 | 7 |
| --- | --- | --- | --- | --- | --- | --- | --- |
| 1. Traditional Paranormal Belief | — |  |  |  |  |  |  |
| 2. New Age Philosophy | .80** | — |  |  |  |  |  |
| 3. Optimism | .16** | .15** | — |  |  |  |  |
| 4. Mental Toughness | -.10** | -.10** | .57** | — |  |  |  |
| 5. Stress | .17** | .14** | -.31** | -.62** | — |  |  |
| 6. Hedonic Well-being | -.06* | -.12** | .48** | .73** | -.50** | — |  |
| 7. Eudaimonic Well-being | .05 | .01 | .59** | .68** | -.39** | .81** | — |

*Note.* **p* < .05, ***p* < .001

Supplementary Table 2. Correlations Among Time 3 Study Variables

| Variable | 1 | 2 | 3 | 4 | 5 | 6 | 7 |
| --- | --- | --- | --- | --- | --- | --- | --- |
| 1. Traditional Paranormal Belief | — |  |  |  |  |  |  |
| 2. New Age Philosophy | .80** | — |  |  |  |  |  |
| 3. Optimism | .11** | .09** | — |  |  |  |  |
| 4. Mental Toughness | -.12** | -.15** | .56** | — |  |  |  |
| 5. Stress | .16** | .19** | -.37** | -.64** | — |  |  |
| 6. Hedonic Well-being | -.08* | -.13** | .50** | .74** | -.51** | — |  |
| 7. Eudaimonic Well-being | .04 | -.01 | .61** | .68** | -.44** | .83** | — |

*Note.* **p* < .05, ***p* < .001

Supplementary Table 3. Standardized Structural Path Estimates for Model 2 (NAP)

| Pathway | β | *p* |
| --- | --- | --- |
| Stability Effects |  |  |
| T1 New Age Philosophy → T2 New Age Philosophy | .82 | < .001 |
| T2 New Age Philosophy → T3 New Age Philosophy | .61 | < .001 |
| T1 New Age Philosophy → T3 New Age Philosophy | .33 | < .001 |
| T1 Optimism → T2 Optimism | .75 | < .001 |
| T2 Optimism → T3 Optimism | .46 | < .001 |
| T1 Optimism → T3 Optimism | .41 | < .001 |
| T1 Mental Toughness → T2 Mental Toughness | .80 | < .001 |
| T2 Mental Toughness → T3 Mental Toughness | .52 | < .001 |
| T1 Mental Toughness → T3 Mental Toughness | .42 | < .001 |
| T1 Stress → T2 Stress | .53 | < .001 |
| T2 Stress → T3 Stress | .33 | < .001 |
| T1 Stress → T3 Stress | .33 | < .001 |
| T1 Hedonic Well-being → T2 Hedonic Well-being | .56 | < .001 |
| T2 Hedonic Well-being → T3 Hedonic Well-being | .41 | < .001 |
| T1 Hedonic Well-being → T3 Hedonic Well-being | .34 | < .001 |
| T1 Eudaimonic Well-being → T2 Eudaimonic Well-being | .79 | < .001 |
| T2 Eudaimonic Well-being → T3 Eudaimonic Well-being | .36 | < .001 |
| T1 Eudaimonic Well-being → T3 Eudaimonic Well-being | .34 | < .001 |
| Cross-Lagged Effects |  |  |
| T1 New Age Philosophy → T2 Optimism | .01 | .552 |
| T2 Optimism → T3 Stress | -.02 | .507 |
| T2 Optimism → T3 Hedonic Well-being | .02 | .502 |
| T2 Optimism → T3 Eudaimonic Well-being | .08 | .021 |
| T2 Mental Toughness → T3 Stress | -.29 | < .001 |
| T2 Mental Toughness → T3 Hedonic Well-being | .39 | < .001 |
| T2 Mental Toughness → T3 Eudaimonic Well-being | -.001 | .953 |

Supplementary Table 3 *continued*

| Pathway | β | *p* |
| --- | --- | --- |
| Direct Effects (T1 → T3) |  |  |
| T1 New Age Philosophy → T3 Stress | .02 | .434 |
| T1 New Age Philosophy → T3 Hedonic Well-being | .001 | .866 |
| T1 New Age Philosophy → T3 Eudaimonic Well-being | .01 | .496 |
| T1 Mental Toughness → T3 Stress | -.18 | < .001 |
| T1 Mental Toughness → T3 Hedonic Well-being | .18 | < .001 |
| T1 Mental Toughness → T3 Eudaimonic Well-being | .14 | < .001 |
| T1 Optimism → T3 Stress | -.04 | .284 |
| T1 Optimism → T3 Hedonic Well-being | .01 | .679 |
| T1 Optimism → T3 Eudaimonic Well-being | .04 | .244 |
| Indirect Effects (T1 → T3 via T2 Variables) |  |  |
| T1 New Age Philosophy → T2 Optimism → T3 Stress | .001 | .767 |
| T1 New Age Philosophy → T2 Optimism → T2 Mental Toughness → T3 Stress | .001 | .566 |
| T1 New Age Philosophy → T2 Optimism → T3 Hedonic Well-being | .001 | .768 |
| T1 New Age Philosophy → T2 Optimism → T2 Mental Toughness → T3 Hedonic Well-being | .001 | .564 |
| T1 New Age Philosophy → T2 Optimism → T3 Eudaimonic Well-being | .001 | .601 |
| T1 New Age Philosophy → T2 Optimism → T2 Mental Toughness → T3 Eudaimonic Well-being | .001 | .976 |

*Note.* Values are standardized coefficients (β). New Age Philosophy was assessed across three time points. All paths are presented in forward temporal order (T1 → T2 → T3). Models were estimated using maximum likelihood with bootstrapped standard errors.
